# Impact of Foliar Application of Chitosan Dissolved in Different Organic Acids on Isozymes, Protein Patterns and Physio-Biochemical Characteristics of Tomato Grown under Salinity Stress

**DOI:** 10.3390/plants10020388

**Published:** 2021-02-18

**Authors:** Mohamed S. Attia, Mahmoud S. Osman, Amr S. Mohamed, Hany A. Mahgoub, Mohamed O. Garada, Eslam S. Abdelmouty, Arafat Abdel Hamed Abdel Latef

**Affiliations:** 1 Botany and Microbiology Department, Faculty of Science, Al-Azhar University, Cairo 11651, Egypt; h.mahgoub@azhar.edu.eg (H.A.M.); drislam20013@gmail.com (E.S.A.); 2 Biotechnology Lab, Horticulture Research Institute, Agricultural Research Center, Giza 12619, Egypt; amr_amz_9@yahoo.com; 3 Research and Development Department, Smart Land for Agriculture Development Co., Giza, Egypt; M.garada19@yahoo.com; 4 Chemistry Department, Faculty of Science, Tanta University, Tanta 31527, Egypt; 5 Department of Biology, Turabah University College, Turabah Branch, Taif University, Taif 21974, Saudi Arabia

**Keywords:** tomato, salinity, antioxidant enzymes, chitosan, organic acids, isozymes, protein pattern, osmolytes

## Abstract

In this study, the anti-stress capabilities of the foliar application of chitosan, dissolved in four different organic acids (acetic acid, ascorbic acid, citric acid and malic acid) have been investigated on tomato (*Solanum lycopersicum* L.) plants under salinity stress (100 mM NaCl). Morphological traits, photosynthetic pigments, osmolytes, secondary metabolites, oxidative stress, minerals, antioxidant enzymes activity, isozymes and protein patterns were tested for potential tolerance of tomato plants growing under salinity stress. Salinity stress was caused a reduction in growth parameters, photosynthetic pigments, soluble sugars, soluble proteins and potassium (K^+^) content. However, the contents of proline, ascorbic acid, total phenol, malondialdehyde (MDA), hydrogen peroxide (H_2_O_2_), sodium (Na^+^) and antioxidant enzyme activity were increased in tomato plants grown under saline conditions. Chitosan treatments in any of the non-stressed plants showed improvements in morphological traits, photosynthetic pigments, osmolytes, total phenol and antioxidant enzymes activity. Besides, the harmful impacts of salinity on tomato plants have also been reduced by lowering MDA, H_2_O_2_ and Na^+^ levels. Chitosan treatments in either non-stressed or stressed plants showed different responses in number and density of peroxidase (POD), polyphenol oxidase (PPO) and superoxide dismutase (SOD) isozymes. NaCl stress led to the diminishing of protein bands with different molecular weights, while they were produced again in response to chitosan foliar application. These responses were varied according to the type of solvent acid. It could be suggested that foliar application of chitosan, especially that dissolved in ascorbic or citric acid, could be commercially used for the stimulation of tomato plants grown under salinity stress.

## 1. Introduction

Salinity considers one of the major abiotic stressors causing severe damage to crops throughout the world. The surge in salinity of the aqueous component of soil will lead to a negative impact on the yield [1]. It is predicted that the affected agricultural land will increase and the problem will get worse as a result of global climate change. All the important physiological and metabolic pathways of plants are affected by salinity [2], besides its effects on nucleic acids (DNA and RNA) and mitosis [3]. Various biological processes in plants are affected as a result of an imbalance in the nutrient content, as well as ionic and osmotic stress, and/or these factors combined as a result of salt stress [4,5]. To overcome the osmotic and ionic stress, plants were able to evolve their biochemical mechanisms such as modulating the osmotic and ionic pressure of cells as well as developing the enzymatic defense mechanism and synthesis of compatible solutes [6]. Obvious oxidative stress markers resulting from high salinity stress are the formation of reactive oxygen species (ROS) such as superoxide ions, hydrogen peroxide, hydroxyl radicals and hydrogen peroxide (H_2_O_2_), which are proven to be highly detrimental to plants [7]. In plants grown under salt stress, substantial elevations of ROS scavenging enzymes such as polyphenol oxidase (PPO), peroxidase (POD), superoxide dismutase (SOD) and catalase (CAT) have been documented [8,9]. In addition to physiological markers of salinity tolerance, both molecular and biochemical markers also show promise in helping tomato screening and breeding phenomena aimed at improving its salinity tolerance. Biochemical markers have provided great interest in recent years as the data more accurately reflect genetic variability since they are direct gene products.

Electrophoretic analysis of total soluble proteins by the sodium dodecyl sulfate (SDS) method and isozyme profiles are valuable in providing a basic need to assess some measures of genetic variability in and among cultivars [10]. The electrophoretically separable variant of the isozymes system is widely used as a biochemical marker, and therefore their analysis can provide a precise tool to discriminate plants grown under saline stress conditions. The identification of isozymes patterns is very important to investigate each isoform activity. Isozyme markers are mostly co-dominant with a simple Mendelian inheritance in most loci and it can be resolved for most plant species regardless of habitat, size, or longevity. The use of sodium dodecyl sulfate-polyacrylamide gel electrophoresis (SDS-PAGE) and isozymes were the simplest and best methods to provide clear information [11,12].

Chitosan is a natural-based linear polysaccharide derived from chitin, the second most abundant biopolymer in nature, and is present as a component in crustacean shells, insect exoskeletons, and fungal cell walls [13]. After the suitable processing of raw chitin, a partial (at least 50%) or complete alkaline deacetylation process is carried out to prepare chitosan. The produced chitosan is composed of glucosamine and N-Acetylglucosamine units. The degree of deacetylation became higher if a larger amount of N-Acetylglucosamine is turned into glucosamine units, which determines its physical properties including solubility, adsorption capability, and biodegradability [14]. Chitosan has two types of functional groups: hydroxyl groups and amino groups, while the functionality of chitosan increases with increasing amino groups [15]. Chitosan induces various defensive responses related to salinity stress in plants [16]. With the shift in climatic conditions and increased food demand leading to inefficient use of synthetic chemicals, the application of chitosan as an elicitor has a large prospect of resolving stress adaptation issues due to abiotic and biotic stresses. In plants, chitosan has been used to develop resistance to abiotic stressors [17]. Their ability to scavenge ROS and eventually enhance stress efficiency has attracted researchers to deliver a more diverse application and continue exploring this new biopolymer. Chitosan at low concentrations could ameliorate the negative impact of salinity stress. The results of [18] showed that the use of low concentration chitosan increased the resistance of safflower and sunflower plants to salt stress by reducing the enzyme activity in these plants. Besides, studies of [19,20,21,22] on *Trachyspermum ammi*, *Plantago ovata*, *Vigna radiata* and *Zea mays*, respectively showed that treatment with chitosan reduced the impacts of salinity on the previous plants by increasing the activity of antioxidant enzymes, which caused a decrease in the malondialdehyde (MDA) content. The intrinsic property of chitosan is that it is not dissolved in neutral aqueous solutions, but rather in acidic solutions of weak carboxylic acids, such as acetic, ascorbic, citric, lactic, and malic acids. Using acetic acid, as it is the associated organic acid to facilitate the dissolution of chitosan, is common in commercial formulations in the agricultural sector. Although acetic acid was reported to be the best associated organic acid in the case of coating fruits to prevent fungal growth [23], the effect of the associated organic acid on plant biostimulant activity was not evaluated. Using other organic acids such as citric and ascorbic acids, which are known for their stimulant activities on plants [24], could lead to synergetic effects, which might increase the performance of the biostimulant. Recently, several researchers demonstrated the stimulating effect of chitosan against abiotic stress on tomato plants [25,26,27,28]. Moreover, [29] reported that foliar application of chitosan ameliorates the negative impact of salinity on tomato plants through enhancing growth aspects and photosynthetic pigments.

Tomato (*Solanum lycopersicum* L.) is a short-lived perennial cropped and is annual. It is part of the Solanaceae (nightshade) family and is usually grown for its edible fruits. Tomatoes are considered among the most important crops grown over the world for their economic and nutritional value [30,31].

This research aims to evaluate and compare the anti-stress capabilities of the foliar application of chitosan, dissolved in four different organic acids (acetic acid, ascorbic acid, citric acid and malic acid) on tomato under salinity stress, suggesting the best solvent that could give synergetic effects with chitosan and incorporate within salinity anti-stress formulations in the commercial sector. It can be said that this is the first investigation into the application of isozymes and protein patterns in determining the impact of chitosan on salinized tomato plants at the molecular level.

## 2. Results

### 2.1. Chitosan Characterization

**Figure 1:**
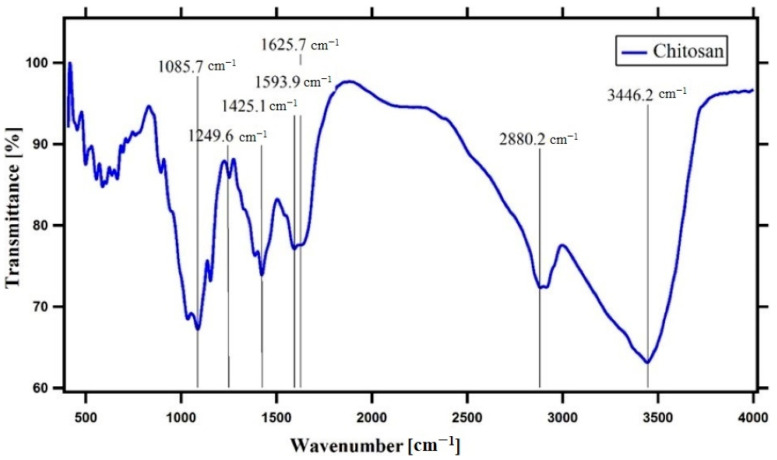
The Fourier transform infrared (FTIR) spectroscopy pattern of the used chitosan sample.

The molecular weight of the used chitosan sample was provided by the manufacturer at a range of 50–150 kDa. The estimated viscosity of 1% chitosan solution in 1% acetic acid at RPM 60 was 12.10 ± 0.2 CP, and at RPM 100 was 14.70 ± 0.4 CP. The DDA was about 65%. The major beaks of chitosan were observed as in Figure 1. A strong band at around 1088 cm^−1^ corresponds to C-O stretching. The absorption band centered on 2880 cm^−1^ can be attributed to C-H asymmetric stretching. The presence of bands at around 1425 and 1382 cm^−1^ may confirm CH_2_ bending and CH_3_ symmetrical deformations, respectively. The absorption band at 1155 cm^−1^ can be attributed to the asymmetric stretching of the C-O-C bridge. The signal at 891 cm^−1^ may be corresponding to the CH bending out of the plane of the ring of monosaccharides. These bands are characteristics of chitosan as reported by others [32]. The presence of N-acetyl groups was confirmed by the bands at around 1626 cm^−1^ (C=O stretching of amide I) and 1594 cm^−1^ (N-H bending of primary amine), respectively. The beak around 1250 cm^−1^ was assigned as the bending vibrations of hydroxyls present in chitosan. 3446 cm^−1^ corresponds to N-H and O-H stretching, as well as the intermolecular hydrogen bonds.

### 2.2. Growth Parameters

**Figure 2:**
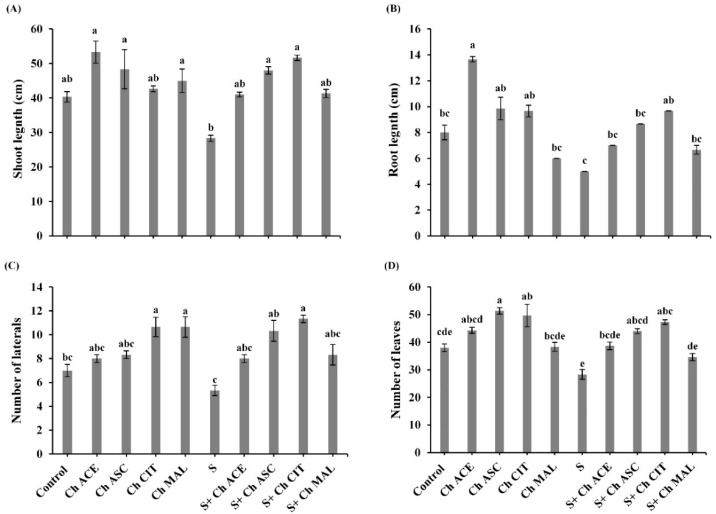
Effect of salinity (S) and foliar application of chitosan dissolved in different organic acids (Ch ACE, Ch ASC, Ch CIT and Ch MAL) and their interactions on (**A**) Shoot length, (**B**) Root length, (**C**) Number of laterals and (**D**) Number of leaves of tomato plants. Data are presented as means ± SE (*n* = 3). Data followed by different letters are significantly different following Tukey’s honestly significant difference (HSD) test at *p* ≤ 0.05.

The results have shown that salinity stress had a depressive effect on all parameters of vegetative growth. In comparison with the control, 100 mM NaCl (S) reduced shoot length by (29.76%), root length by (37.50%), number of laterals by (23.86%), and number of leaves by (25.45%), respectively (Figure 2). Concerning the impact of foliar application chitosan solutions on the stressed plants, it was noticed that application with (Ch CIT and Ch ASC) boosted shoot length by (82.35% and 69.43%), root length by (93.20 and 73.20%), number of lateral by (112.75% and 93.80%) and number of leaves by (67.06% and 55.31%), respectively, and came next treatment with (Ch ACE and Ch MAL) which recorded a marked improvement in shoot length by (44.72% and 45.88%), root length by (40% and 33.20%), number of lateral by (50.09% and 56.28%), and number of leaves by (36.46% and 22.13%), respectively versus stressed plants. Unstressed tomato plants treated with chitosan solutions (Ch ACE, Ch ASC, Ch CIT and Ch MAL) showed an improvement in morphological aspects shoot length by (32.23%, 19.83%, 5.77% and 11.57%), root length by (70.75%, 23.25%, and 20.75%), number of laterals by (14.28%, 19%, 52.28% and 52.28%), and number of leaves by (15.56%, 33.96%, 29.55% and 0.87%), respectively over control plants.

### 2.3. Photosynthetic Pigments

**Figure 3:**
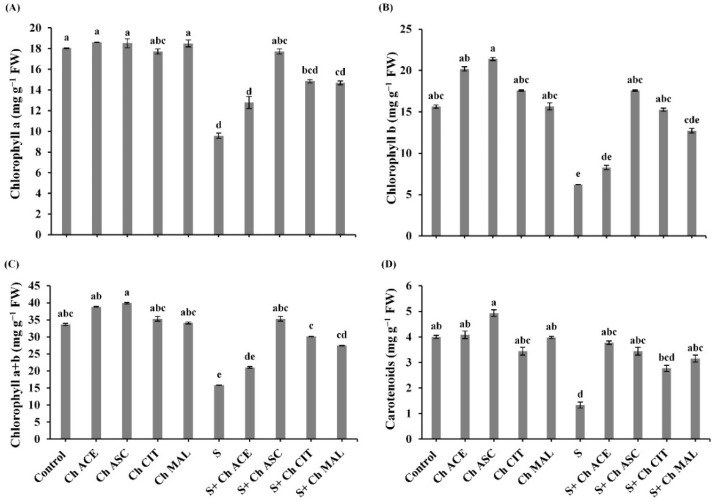
Effect of salinity (S) and foliar application of chitosan dissolved in different organic acids (Ch ACE, Ch ASC, Ch CIT and Ch MAL) and their interactions on (**A**) Chlorophyll a, (**B**) Chlorophyll b, (**C**) Chlorophyll a+b and (**D**) Carotenoids of tomato plants. Data are presented as means ± SE (*n* = 3). Data followed by different letters are significantly different following Tukey’s honestly significant difference (HSD) test at *p* ≤ 0.05. FW: fresh weight.

The contents of chlorophyll a, b as well as chlorophyll a+b were markedly decreased in stressed plants (Figure 3). However, stressed plants treated with chitosan solutions (Ch ACE, Ch ASC, Ch CIT and Ch MAL) showed a marked increase over untreated stressed plants. Concerning the effect of chitosan solutions (Ch ACE, Ch ASC, Ch CIT and Ch MAL) on the challenged plants with NaCl, it was found that (Ch ASC and Ch CIT) showed a marked increase in the contents of chlorophyll a by (85.26% and 55.17%), chlorophyll b by (182.02% and 144.49%) and chlorophyll a+b by (123.33% and 90.51%), respectively, while application of (Ch ACE and Ch MAL) recorded a noticeable improved in the contents of chlorophyll a by (33.64% and 53.50%), chlorophyll b by (32.58% and 104.33%) and chlorophyll a+b by (33.14% and 73.43%), respectively against NaCl stressed plants (Figure 3). The unstressed tomato plants treated with chitosan solutions (Ch ACE, Ch ASC, Ch CIT and Ch MAL) showed increases in the content of chlorophyll b by (29.17%, 36.85%, 12.41 and 0.19%) and chlorophyll a+b by (15.29%, 18.56%, 4.87% and 1.18%), respectively. While the content of chlorophyll a insignificantly increased in response to the application of chitosan solutions. In plants exposed to the salinity stress content of carotenoids decreased by 66.75% when being compared with S plants only. Moreover, the obtained results illustrated that in salinized plants, the content of carotenoids was increased in response to the treatment with chitosan solutions (Ch ACE, Ch ASC, Ch CIT and Ch MAL) plants only (Figure 3).

### 2.4. Osmolytes

Salt-stressed tomato plants showed decreases in contents of soluble sugars and soluble proteins by 15% and 36.76%, respectively (Table 1). However, the content of proline was increased significantly by 27.55% as compared to control plants (Table 1). Foliar application of chitosan dissolved in different solutions enhanced the contents of tested osmolytes in shoots of salinity-stressed tomato plants over S plants only. The highest recorded increase in contents of soluble sugars, soluble proteins, and proline content was noticed in Ch ASC by 27.36%, 112.24% and 46.29%, respectively over salinity-stressed plants. Application of chitosan dissolved in different organic acids (Ch ACE, Ch ASC, Ch CIT and Ch MAL) alone elevated soluble sugars by (17.91%, 33.41%, 23.26% and 34.48) and soluble proteins by (42.29%, 51.46%, 42.74% and 46.06%), respectively over control plants (Table 1). The highest recorded increase in contents of soluble sugars and soluble proteins in response to foliar application of chitosan alone was in the case of Ch ASC, Ch MAL, Ch CIT, and finally Ch ACE, respectively.

### 2.5. Phenols and Ascorbic Acid

**Figure 4:**
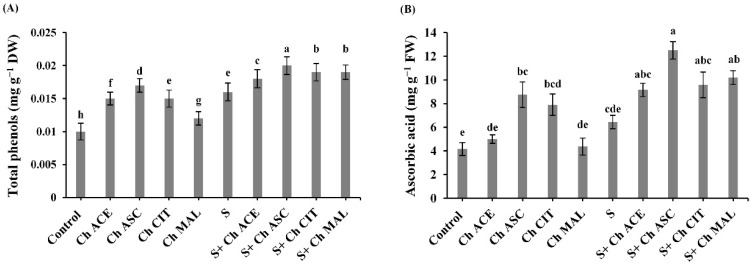
Effect of salinity (S) and foliar application of chitosan dissolved in different organic acids (Ch ACE, Ch ASC, Ch CIT and Ch MAL) and their interactions on the content of (**A**) Total phenols and (**B**) Ascorbic acid of tomato plants. Data are presented as means ± SE (*n* = 3). Data followed by different letters are significantly different following Tukey’s honestly significant difference (HSD) test at *p* ≤ 0.05. FW: fresh weight, DW: dry weight.

Tomato plants grown under NaCl stress regimes exhibited significant increases in contents of total phenols and ascorbic acid by 60% and 55.04%, respectively versus control plants (Figure 4). Moreover, foliar application of chitosan with different solutions resulted in a noticeable increase in the content of total phenols by 12.50%, 25%, 18.75%, and 18.75% at Ch ACE, Ch ASC, Ch CIT and Ch MAL, respectively, and in the content of ascorbic acid by 42.01%, 93.79%, 48.52% and 58.13% at Ch ACE, Ch ASC, Ch CIT and Ch MAL, respectively over S plants only. The highest increase in contents of total phenols and ascorbic acid was recorded in Ch ASC-treated plants (Figure 4). Under control conditions, chitosan-treated tomato plants (Ch ACE, Ch ASC, Ch CIT and Ch MAL) showed an improvement in total phenol content by (50%, 70%, 50% and 20%) and ascorbic acid content by (20.19%, 110.33%, 90.14% and 5.04%), respectively.

### 2.6. Oxidative Stress

**Figure 5:**
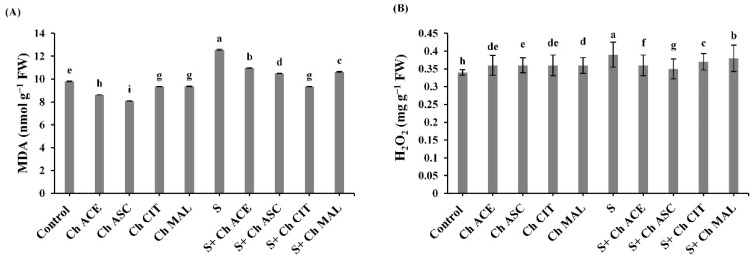
Effect of salinity (S) and foliar application of chitosan dissolved in different organic acids (Ch ACE, Ch ASC, Ch CIT and Ch MAL) and their interactions on (**A**) Malondialdehyde (MDA) and (**B**) Hydrogen peroxide (H_2_O_2_) of tomato plants. Data are presented as means ± SE (*n* = 3). Data followed by different letters are significantly different following Tukey’s honestly significant difference (HSD) test at *p* ≤ 0.05. FW: fresh weight.

Salinity stress accumulated the contents of MDA and H_2_O_2_ by 28.39 and 14.70%, respectively compared to unstressed tomato plants (Figure 5). The content of MDA was declined in response to different chitosan treatments by 12.81, 16.55, 25.70 and 15.44 at Ch ACE, Ch ASC, Ch CIT and Ch MAL, respectively. While the content of H_2_O_2_ was reduced by 7.70, 10.25, 5.12 and 2.56% at Ch ACE, Ch ASC, Ch CIT and Ch MAL, respectively compared to salinized tomato plants (Figure 5).

### 2.7. Sodium (Na^+^) and Potassium (K^+^) Contents

Under salinity stress conditions tomato plants showed significant increases in Na^+^ content by 98.84%. While K^+^ content was significantly decreased in tomato shoots by 48.86% when compared to control plants (Table 2). Moreover, foliar application of chitosan dissolved in different organic acids resulted in a remarkable decrease in the Na^+^ content by 48.94%, 41.79%, 51.52% and 43.45% at Ch ACE, Ch ASC, Ch CIT and Ch MAL, respectively. However, K^+^ content was increased by 20.05%, 30.08%, 10.66% and 21.47% at Ch ACE, Ch ASC, Ch CIT and Ch MAL, respectively when compared with salinized tomato plants (Table 2). Under control conditions, foliar application of chitosan dissolved in different organic acids (Ch ACE, Ch ASC, Ch CIT and Ch MAL) induced a noticeable improvement in K^+^ content by (54.22%, 65.49%, 16.19% and 75.35), respectively.

### 2.8. Antioxidant Enzymes Activity

**Figure 6:**
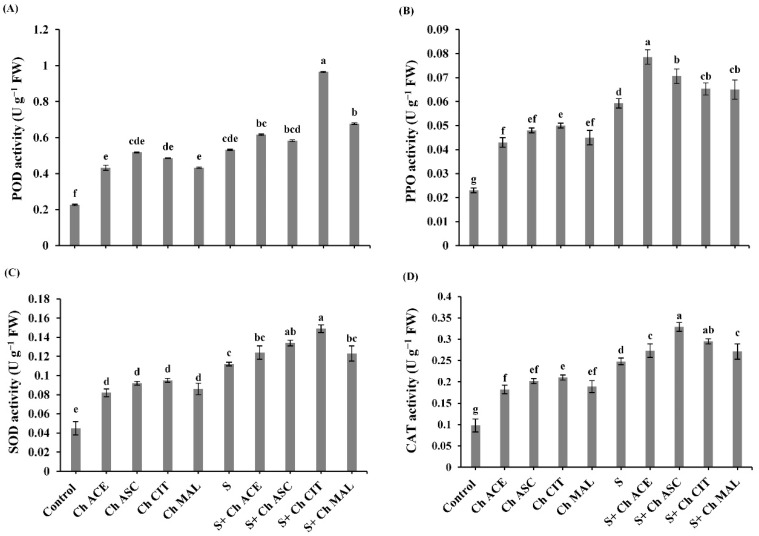
Effect of salinity (S) and foliar application of chitosan dissolved in different organic acids (Ch ACE, Ch ASC, Ch CIT and Ch MAL) and their interactions on (**A**) Peroxidase (POD) activity, (**B**) Polyphenol oxidase (PPO) activity, (**C**) Superoxide dismutase (SOD) activity and (**D**) Catalase (CAT) activity of tomato plants. Data are presented as means ± SE (*n* = 3). Data followed by different letters are significantly different following Tukey’s honestly significant difference (HSD) test at *p* ≤ 0.05. FW: fresh weight.

The activity of SOD, CAT, POD and PPO was boosted in salinity stress tomato plants compared to untreated control plants (Figure 6). Moreover, spraying of chitosan solutions (Ch ACE, Ch ASC, Ch CIT and Ch MAL) increased the activity of SOD by (16.78%, 10.07%, 24.84% and 17.45%), CAT by (16.77%, 24.4%, 10.07% and 17.38%), POD by (36.17%, 39.59%, 46.33% and 29.75%) and PPO by (16.67%, 10.26%, 24.36% and 16.67%), respectively, over salinized plants (Figure 6). Under non-stressed conditions, foliar application of chitosan dissolved in different organic acids (Ch ACE, Ch ASC, Ch CIT and Ch MAL) boosted the activities of SOD, CAT, PPO and POD.

### 2.9. Isozymes

#### 2.9.1. POD Isozymes

**Figure 7:**
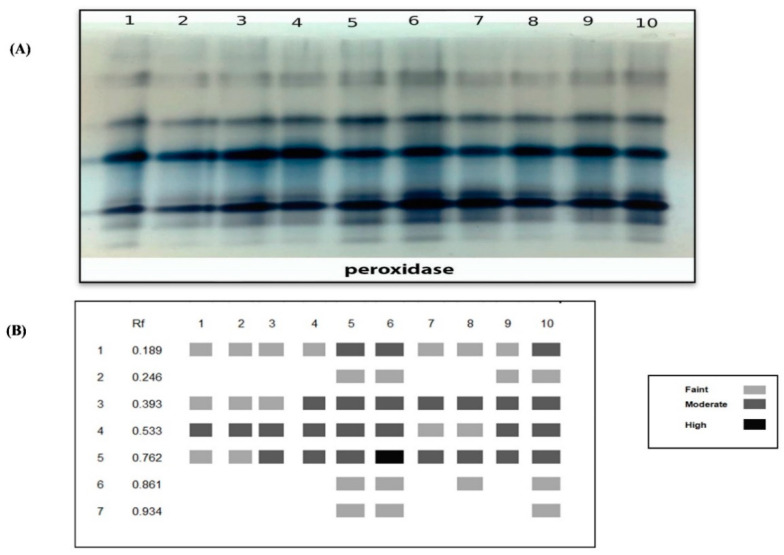
Effect of salinity (S) and foliar application of chitosan dissolved in different organic acids (Ch ACE, Ch ASC, Ch CIT and Ch MAL) and their interactions on (**A**) Peroxidase isozyme and (**B**) Ideogram analysis of peroxidase isozyme of tomato plants.

Native PAGE in Figure 7 and Table 3 showed seven POD isozymes at Rf (0.189, 0.246, 0.393, 0.533, 0.762, 0.861 and 0.934). Salinity-stressed plants showed highly overexpressed POD that recorded 7 bands including 3 faint bands at Rf (0.246, 0.861 and 0.934), 3 moderate bands at Rf (0.189, 0.393 and 0.533) and 1 highly dense band at Rf (0.762). Under the saline conditions, chitosan foliar application (Ch MAL) recorded the same 7 bands at the same Rf in which 4 of them were moderated bands at Rf (0.189, 0.393 and 0.762), while the other 4 bands were faint at Rf (0.246, 0.533, 0.861 and 0.934) followed by (Ch CIT) treatment that showed 5 bands, 3 of them moderate at Rf (0.393, 0.533 and 0.762). Untreated control plants expressed the lowest POD expression that they produced 3 faint bands at Rf (0.189, 0.393 and 0.762) and 1 moderate band at (0.533).

#### 2.9.2. PPO Isozymes 

**Figure 8:**
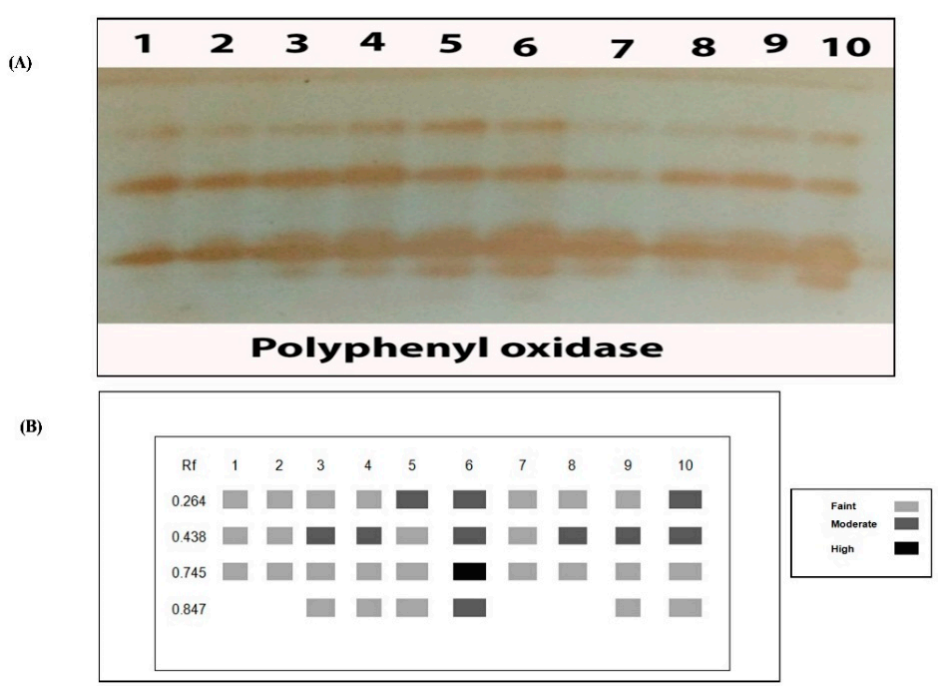
Effect of salinity (S) and foliar application of chitosan dissolved in different organic acids (Ch ACE, Ch ASC, Ch CIT and Ch MAL) and their interactions on (**A**) Polyphenol oxidase isozyme and (**B**) Ideogram analysis of polyphenol oxidase isozyme of tomato plants.

The PPO isozyme of tomato plant leaves showed four PPO isozymes at Rf (0.264, 0.438, 0.745 and 0.847) in Figure 8 and Table 4. Salinity-stressed plants showed the highly PPO expression that produced 4 bands including three moderate bands at Rf (0.264, 0.438 and 0.847), 1 highly dense band at Rf (0.745). However, control plants showed the lowest PPO that produced three faint bands at the same Rf (0.264, 0.438 and 0.745). Under salt stress conditions chitosan treatment (Ch ASC and Ch CIT) recorded 3 faints bands at Rf (0.264, 0.745 and 0.847) and 1 moderate band at Rf (0.438). While Ch MAL treatment gave a high expression of PPO but was lower than salinity-stressed plants resulted in 2 moderate bands at Rf (0.264 and 0.438) and 2 faint bands at Rf (0.745 and 0.847).

#### 2.9.3. SOD Isozymes

**Figure 9:**
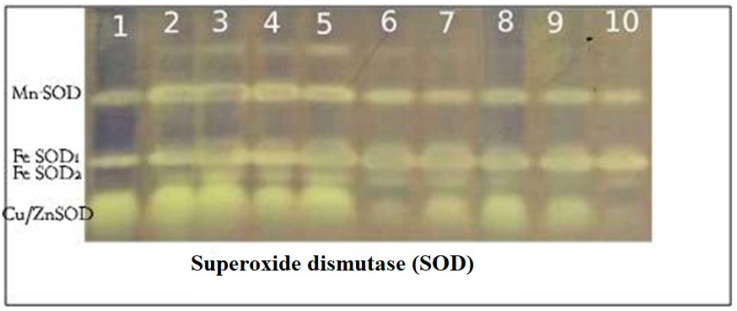
Effect of salinity (S) and foliar application of chitosan dissolved in different organic acids (Ch ACE, Ch ASC, Ch CIT and Ch MAL) and their interactions on Superoxide dismutase (SOD) isozyme of tomato plants.

The results in Table 5 and Figure 9 showed that tomato plants expressed four SODs (Mn SOD, Fe SOD1, Fe SOD2 and Cu/Zn SOD) at Rf (0.362, 0.641, 0.727 and 0.865), respectively. Salinized tomato plants showed Cu/Zn SOD faint band at Rf (0.865). However, chitosan treatments showed Cu/Zn SOD band in the range between moderate and high density. Tomato leaves expressed Fe SOD1 band at Rf (0.641), which present in treated and untreated plants. However, FeSOD2 at Rf (0.727) was absent from untreated control plants and was present in control and salinity-stressed tomato plants. Mn SOD detected at Rf (0.362) in tomato plants leaves had a high density of S + Ch ACE, S + Ch ASC and S + Ch CIT and then S, S + Ch MAL, respectively.

### 2.10. SDS-PAGE

**Figure 10:**
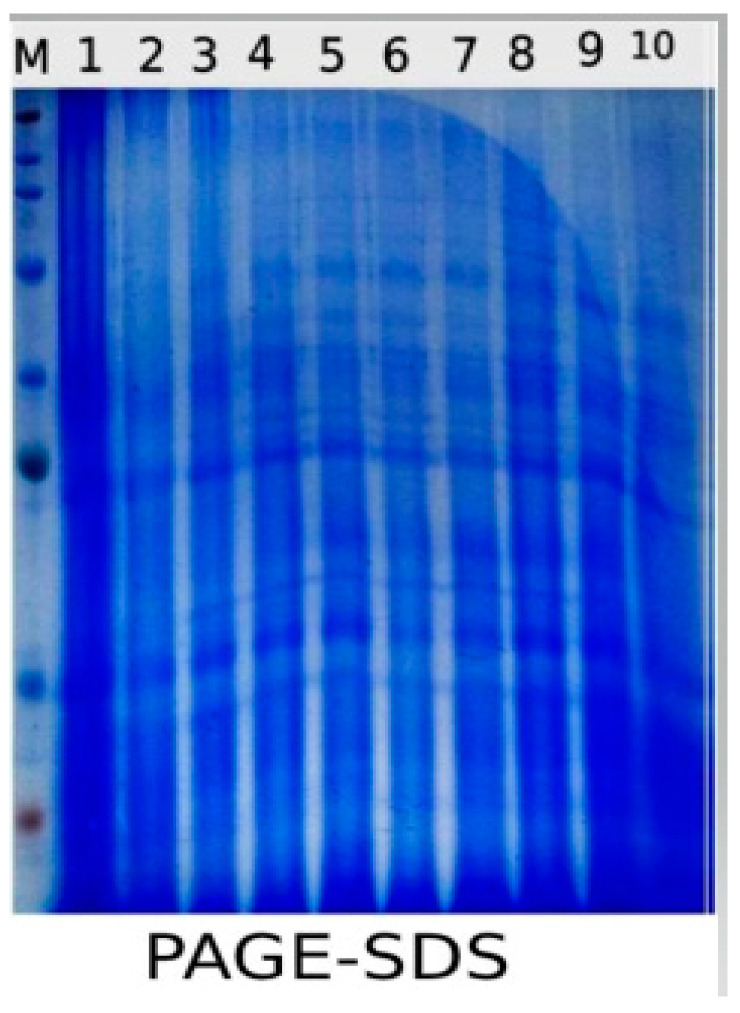
Effect of salinity (S) and foliar application of chitosan dissolved in different organic acids (Ch ACE, Ch ASC, Ch CIT and Ch MAL) and their interactions on Protein electrophoretic banding patterns of tomato leaves. L1 = Control, L2 = Ch ACE, L3 = Ch ASC, L4 = Ch CIT, L5 = Ch MAL, L6 = S, L7 = S + Ch ACE, L8 = S + Ch ASC, L9 = S + Ch CIT and L10 = S + Ch MAL.

The protein banding profiles of the 10 treatments as revealed by SDS-PAGE are illustrated in (Figure 10) and (Table 6). The total number of bands was 10 with molecular weights ranging from 15.456 to 116.221 KDa. The highest number of bands was 9, detected in Ch ASC, Ch CIT and S + Ch ASC, while the lowest number of bands was 7, identified in S + Ch MAL, while Control, Ch ACE, Ch MAL, S, and S + Ch AC recorded 8 bands. Demonstrative analysis of the presence and absence of bands was assessed with (+) and (−), respectively, as illustrated in Table 6. Our results showed the disappearance of the polymorphic band with molecular weight 72.115 KDa in S plants as compared to control plants. The aforementioned band reappeared again in response to chitosan application S + ACE, S + ASC and S + Ch CIT. Moreover, it is observed that the diminishing of one band with molecular weight 85.359 KDa in S plants while appeared again in S + ACE and S + ASC. Furthermore, another polymorphic band with a molecular weight 28.165 kDa disappeared in S plants while it appeared again in S + ACE, S + ASC and S + Ch CIT.

## 3. Discussion

Salinity stress is considered one of the most critical challenges facing countries, especially Egypt. The growing knowledge of environmental problems, therefore, makes it important to seek alternatives that are easy to use and feasible to overcome the harmful impacts of salinity on plants [33,34,35]. Morphological aspects (shoot length, root length, number of laterals branches per plant and number of leaves) were significantly decreased due to salt stress. In this regard, the reduction in growth may be correlated with different factors; among them are high osmotic stress and ion toxicity [9,36,37]. The first standard to govern the occurrence of tolerance in tomato plants, foliar application with chitosan solutions was the enhancement of growth parameters. Reports have shown that the application of inducers such as chitosan improved morphological characteristics in the case of maize [38,39], rice [40,41], and common beans [42] and stimulate tolerance of seedlings under stress conditions. Thus, the use of chitosan dissolved in some acids, which are low-molecular-weight organic acids such as citric acid, ascorbic acid and malic acids improves the plant’s ability to ameliorate abiotic stress [16]. The chitosan solutions used in this study were not phytotoxic. Plants treated with chitosan dissolved in acetic acid have not shown phytotoxicity in different crops such as Japanese pear [43], kiwifruit, or table grape [44]. Concerning interaction effects, foliar application of chitosan enhanced morphological aspects of tomato plants. These stimulating effects of chitosan were clear due to the presence of ascorbic acid and citric acid as antioxidants.

Organic acids such as ascorbic acid, citric acid, acetic acid, and malic acid boosted the growth of different plants as they enhanced the photosynthetic process throughout increasing chlorophyll contents. Moreover, they play a major role in abolishing the adverse effects of abiotic stresses, protecting protein and lipid, increasing proline contents, and decrease lipid peroxidation [45,46,47,48,49,50].

Photosynthetic pigments were clear positive evidence as a result of the application of the chitosan solutions and became a visible piece of evidence of sufficient treatments. In the current study, the results clearly showed a lessening in the photosynthetic pigment levels in the leaves of tomato plants due to salt stress. The decrease in photosynthetic pigments may be due to a deficiency in the leaf area responsible for light capture and photosynthesis, or may also be due to the degradation of chlorophyll by increasing the activity of chlorophyll degrading enzymes and chlorophyllase under salt stress regimes [34,36]. On the contrary, data in the current study have shown that treatment of tomato plants grown under salinity stress conditions then treated with chitosan solutions significantly improved plant salt tolerance by increasing photosynthetic pigments. It was stated in [51] that the application of chitosan enhanced photosynthetic rates of *Oryza sativa* plants throughout enhancement photosynthetic pigments. This augmentation might be attributed to improved stomatal conductance, transpiration rate and/or cell size and number [52]. This may also be since chitosan has been reported to cause plant defense reactions [53], and it may trigger NADPH oxidase activity, thereby activating the production of H_2_O_2_. Thus, chitosan could activate ROS scavenging systems in plants [54].

The accumulation of osmolytes serves as a common phenomenon that plays an important role in ROS scavenging, supply plant cells with energy as well as modulating cell redox homeostasis [9,55,56]. In this work, there is a positive correlation between the reduction in osmolytes contents (soluble sugars and soluble proteins) and a reduction in photosynthetic pigments and the growth of tomato plants in response to salinity stress. However, the content of proline was increased due to its role in osmoregulation and ROS scavenging [57,58]. Foliar application of chitosan dissolved in different organic acids, especially ascorbic acid, enhanced osmolytes in the shoots of tomato plants. These results are in harmony with [59]. A study by [60] stated that a significant increase in osmolyte contents in chitosan-treated milk thistle (*Silybum marianum* L.) plants. Chitosan caused an enhancement in the contents of soluble sugars, soluble proteins throughout its role in increasing the expression of enzymes involved in glycolysis [61,62]. Proline accumulation in tomato shoots prevents the photosynthetic process throughout, preventing damage of photosynthetic pigments caused by ROS [57,63].

ROS scavenging in plants occurs in two ways enzymatically and non-enzymatically to prevent plant cells from oxidative damage. Non-enzymatic pathways include phenolic compounds and ascorbic acid, which can overcome ROS production [9,64,65]. In this study, salinity stress increased the content of total phenols and ascorbic acid in the shoots of tomato plants. Our results are in accordance with other investigators [66,67,68]. Phenolic compounds and ascorbic acid support antioxidant roles by scavenging the free radicals, reducing their reactivity to the membrane components [9,48]. Moreover, Phenolic compounds are also able to stabilize cell membranes by lowering membrane fluidity, which results in reduced mobility of free radicals across membranes, thus limiting membrane peroxidation [65]. Concerning the interaction effect of chitosan dissolved in different organic acids, especially the ascorbic acid foliar application of chitosan, enhanced total phenols and ascorbic acid contents over salinity-stressed plants. The aforementioned increases in ascorbic acid and total phenol contents are in correlation with the reduction in contents of MDA and H_2_O_2_. The accumulation of phenolic compounds and ascorbic acid serves as an adaptive strategy for salinity stress [69,70]. The obtained results are in line with [71,72]. Moreover, a study of [73] indicated that treatment with chitosan increased significantly the content of phenolic compounds, which directly declined lipid oxidation throughout, transferring a phenolic hydrogen atom to a radicle. Moreover, [74] reported the stimulatory role of chitosan on secondary metabolites as phenolic compounds through inducing certain genes involved in the biosynthesis of secondary metabolites.

Oxidative stress caused by salinity stress led to serious disruption to plant cells and increased the contents of MDA and H_2_O_2_ in the leaves of tomato plants. These findings are in harmony with [34,75,76]. Application of chitosan lessened the production of MDA and H_2_O_2_ peroxide through increasing antioxidant compounds that scavenge ROS and prevent cellular membranes from oxidative stress [38]. Moreover, [73,77] stated that chitosan application significantly reduced the contents of MDA in salinity-stressed wheat plants. The protective role of chitosan was more obvious due to the presence of different organic acids which help tomato plants diminish the harmful impact of salt stress [47,48,78].

Salinity-induced growth deficits in crops are mainly associated with ion stress, which arises due to long exposure to salt stress [6,9]. Ionic stress occurs in response to the accumulation of sodium in plant cells, which caused plant toxicity and disrupt normal metabolism of salinity-stressed plants [75,79]. Our results showed an increase in sodium content in tomato shoots; however, potassium content was significantly decreased. These results explain the deleterious effect of sodium accumulation in the plant cell. Chitosan foliar application was reduced sodium accumulation and increase K level in tomato shoots, which caused homeostasis, which is generally observed in salt-tolerant varieties [79,80]. This change in Na^+^ and K^+^ level could be attributed to the ability of chitosan in improving the growth of tomato plants or throughout osmolytes content increases, which acquire a plant balance in facing salinity stress. These results are in harmony with the results of [71,81], who also reported that chitosan treatment significantly increased Na^+^ and K^+^ content in salinity stress wheat plants.

Antioxidant enzymes SOD, CAT, POD and POO provide a large number of defensive enzymes associated with salinity stress [36,82]. These enzymes act as initial steps in increasing plant resistance to various stresses as well as the formation of phenolic compounds [83,84]. The results showed that antioxidant enzyme activity increased in plants exposed to salt stress. The plants show different mechanisms to cope with salinity pressure as they increase the activity of certain antioxidant enzymes to keep ROS at the lower level in the cell. SOD helps in the conversion of O_2_^−^ to H_2_O_2_, which acts as the first line in facing oxidative stress, while CAT and POD help in the conversion of H_2_O_2_ to H_2_O [64]. SOD, CAT, POD and PPO activities were greater in the plants grown under NaCl stress and treated with chitosan solutions against salinized plants. Application of chitosan was reported to increase the activity of catalase and peroxidase in tomato [85], eggplants [86], and milk thistle [60]. The chitosan chemical constitution includes uridine diphosphate N-acetyl-d-glucosamine (UDP-GlcNAc) as nucleotide sugars, which, when applied in plants recognized by cells throughout chitin synthase chitin deacetylase enzymes, caused the formation of chitosan oligomers that are involved in plant cell signals [87,88]. Chitosan oligomers enter the nucleus and act in cascade reactions as the production of hormones and the expression of antioxidant enzymes [62,87,89,90].

Multiple enzyme isoforms are considered a key control mechanism for cell metabolism in plants, and changes in isozyme profiles play an important role in cellular protection versus salt stress [91,92]. The induction of these isozymes is considered to constitute an important role in the cellular defense against oxidative stress [93]. Activity staining of antioxidants after PAGE showed seven POD isozymes, four PPO isozymes and four SOD isozymes in the extract of leaf-soluble proteins (one Mn-SOD, two Fe-SODs, and one CuZn-SOD of tomato plants). In general, the antioxidant enzyme activities in salt-stressed plants treated with chitosan were always higher than those in control plants, because many new isozyme bands were induced by salt stress. Our results have shown that stressed tomato plants treated with chitosan either dissolved in ascorbic acid or citric acid appeared a maximum banding of POD isozyme compared to other treatments. These results reflecting the ameliorative role of chitosan treatments (Ch ASC and Ch CIT) in protecting tomato plants from salt stress. For the PPO isozyme pattern, the present analysis showed that there is no variation in the expression level of PPO isoforms among tomato sprayed with different chitosan treatments either alone or in combination with salinity stress. The only difference was observed for the PPO isozyme pattern in tomato treated with NaCl alone versus control plants. Indeed, the separation of SOD isozymes (after native PAGE) coupled with different specific inhibitors showed four SOD isozymes in the extract of leaf-soluble proteins: one Mn-SOD, two Fe-SODs (denominated Fe-SOD1 and Fe-SOD2), and one Cu/Zn-SOD. Quantification of the SOD band intensities revealed that Fe- SODs and Cu/Zn-SOD were the predominant isozymes. These results are in harmony with [94] who reported enhanced Mn-SOD and Cu/Zn-SOD transcript abundances in maize and tomato plants. The intensity level of all SOD isozymes increased under chitosan treatments as compared to control untreated and salt-treated plants. The highest intensity level of SOD isozymes in tomato leaves was detected with Ch ASC alone. However, the lowest intensity level of SOD isozymes was observed in plants treated with NaCl alone and S + Ch MAL. The results are in line with [95] who noticed that treating tomato plants with NaCl suppressed mRNA levels of SOD genes, whereas plants treated with zinc oxide nanoparticle inducers, in the presence of NaCl, showed an increase in mRNA expression levels, suggesting the beneficial impact of zinc oxide on plant metabolism under salt stress. Similarly, the present data indicate that chitosan, especially when dissolved in ascorbic acid had a positive response on tomato metabolism under salinity stress. The increments of Fe-SODs isozymes could be attributed to their abundance in chloroplasts of tomato plants under investigation [96]. Furthermore, [97] found that overexpression of Cu/Zn SOD in potato showed that transgenic plants exhibited increased tolerance to oxidative stress. The present data were found to agree with previous studies which reported that a variety of protein functions could act as scavengers for these ROS including CAT and POD [93,98]. Our results suggested that the differential responses of tomato plants to NaCl stress depended on the solvent type that chitosan was dissolved in. Thus, we could mention that chitosan dissolved in ascorbic acid (Ch ASC) and/or citric acid (Ch CIT), solvents appeared to have a higher tolerant level against salt stress against other treatments.

SDS-PAGE results illustrated differences in patterns of protein changes between chitosan treatments either alone or in combination with salinity stress and represented protein banding patterns with different molecular weight as an appositive marker and showed more changes in protein profile and a higher percentage of polymorphism in plants treated with chitosan dissolved in Ch ASC or chitosan dissolved in Ch CIT versus other chitosan treatments. The explanation for these findings is that tolerant cultivars are capable of adapting successfully to saline regimes by modifying their biochemical processes and, consequently, by accumulation or depletion of certain metabolite activities, which caused the suppression of a pre-existing protein synthesis and improved or de novo synthesis of proteins which induce resistance strategies. This explanation is also supported by previous results [99,100]. The protein band at molecular weight 72.115 kDa in control plants and salinity-stressed tomato plants treated with chitosan dissolved in acetic acid, ascorbic acid and citric acid can be considered as positive markers for stress, and it was noted that this band was diminished under salinity stress and induced again under the interaction between salinity and chitosan treatments except malic acid-treated plants.

## 4. Materials and Methods

### 4.1. Characterization of Chitosan

Chitosan, with MW 50–150 kDa, has been purchased from a local provider in National Research Center, Giza, Egypt. The organic acids used were purchased from Sigma Aldrich (Merk). All chemicals were used without further purification. Fourier transforms infrared (FTIR) analysis was carried out to investigate the functional groups of the chitosan sample using an FTIR spectrometer (Thermo Scientific Nicolet iN10, national research center, Cairo, Egypt). The measurements were performed by the potassium bromide (KBr) disc method at room temperature within a spectral range of 400–4000 cm^−1^, the number of scans was 16, and the spectral resolution was 4 cm^−1^. The degree of acetylation (DA) of chitosan was estimated according to [101] based on the FTIR spectra. DA was calculated using the absorbance ratio (A1655/A3450) by the following equation: DA (%) = (A1655/A3450) ×100/1.33 and the deacetylation was calculated via subtraction of DA value out of 100% according to the following equation: Degree of deacetylation (%) = 100 − DA (%). The viscosity of 1% chitosan solution in 1% acetic acid was measured using Brookfield RVDVE230 Medium-range viscometer at room temperature. The measurements were carried out in a 250 mL beaker at two rotation per minute (RPM) values, 60 and 100 rpm. The torque values were then converted to centipoise (cp), three cp measurements at each RPM value were collected.

### 4.2. Pot Experiment

Four weeks of age tomato seedlings (cultivar 023) were collected from the Agricultural Research Center (ARC), Giza, Egypt. Uniform seedlings were transplanted into plastic pots (40 cm in diameter) contain a mixture of sand and clay (1:3 W/W), a total of 7 kg, in a plastic greenhouse, at Smart Land company for agriculture development, 6th of October, Egypt. The pots were coordinated in a completely randomized design with six replicates to study the effect of a single variable, the type of organic acid used for the chitosan solution. Pots were arranged as follows: i- Water irrigation group includes Control, Acetic acid Chitosan (Ch ACE), Ascorbic acid chitosan (Ch ASC), Citric acid chitosan (Ch CIT) and malic acid chitosan (Ch MAL) and ii- 100 mM NaCl irrigated group includes: Salinity (S), Acetic acid Chitosan (S + Ch ACE), Ascorbic acid chitosan (S + Ch ASC), Citric acid chitosan (S + Ch CIT) and malic acid chitosan (S + Ch MAL). For all treatments, the concentration of the chitosan and the associated carboxylic acids was 100 ppm and pH 6. There was no mixing, but rather the chitosan is dissolved in each of the four different solutions separately. After the transplant, the seedlings left for five days before any treatments. Afterward, a saline solution (100 mM NaCl) was applied three times (1 time every 5 days). Pots of the control group were irrigated at field capacity with 1000 mL of distilled water and the salinity group pots were irrigated with an equal volume of 100 mM NaCl. After 4 days of NaCl treatment, chitosan was foliar applied 3 times (1 time each week) (in the period before and after flowering) with a range of (400 mL/pot). The samples were collected for different growth traits (shoot length, root length, number of leaves, and number of lateral branches per plant) and biochemical analysis after four weeks from transplanting.

### 4.3. Photosynthetic Pigment Determination

A former procedure mentioned in the study [102] was used to assess the existence of chlorophyll a, chlorophyll b and chlorophyll a + b and carotenoids in fresh tomato leaves. Throughout this procedure, 50 mL of acetone (80%) has been used for photosynthetic pigments extraction from fresh leaves (0.5 g) and the extract was filtered and the developed green color was measured spectrophotometrically at 665, 649 and 470 nm.

### 4.4. Determination of the Content of Osmolytes

The soluble sugar content of the dried shoot was calculated by the method described by [103]. For soluble sugars extraction, the dried shoots (0.5 g) were mixed with 2.5 mL of 2% phenol and 5 mL of 30% trichloroacetic acid then filtrate throughout filter paper; 1 mL of the filtered was then mixed with 2 mL of anthrone reagent (2 g anthrone/L of 95% H_2_SO_4_). The developed blue-green color was measured at 620 nm. The procedure [104] was used to determine the soluble proteins content of the dry shoot. In this method, dried tomato shoots were extracted in 5 mL of 2% phenol and 10 mL of distilled water; 1 mL of extract was mixed with 5 mL of alkaline reagent (50 mL of 2% sodium carbonate prepared in 0.1 N NaOH and 1 mL of 0.5% copper sulfate was prepared in (1% potassium sodium tartrate) and was thoroughly combined with 0.5 mL of Folin’s reagent (diluted 1:3 v/v). The color formed after 30 min was measured at 750 nm. The proline content was measured in the dry shoot according to [105]. In this procedure, the dried shoots (0.5 g) were digested to 10 mL (3%) of sulfosalicylic acid. Two milliliters of filtrate reacted with 2 mL of ninhydrin acid (1.25 g ninhydrin in 30 mL of glacial acetic acid and 20 mL of 6 M phosphoric acid) and 2 mL of glacial acetic acid in a boiling water bath for 1 h, then the reaction was stopped by placing in an ice bath. We added 4 mL of toluene to the mixture, then read the absorbance at 520 nm.

### 4.5. Determination of Ascorbic Acid and Total Phenol Contents

The technique of [106] was used to estimate the ascorbic acid content of the fresh shoot. Shoot samples (0.5 g) were ground with liquid nitrogen and suspended in 2 mL of 5% trichloroacetic acid (TCA) and then centrifuged at 10,000× *g* rpm for 15 min at 4 °C. Two milliliters of supernatant was mixed with 8 mL of 10% TCA. After intensive shaking, the samples were kept in an ice bath for 5 min and centrifuged at 3000× *g* rpm for another 5 min; 5 mL of the extract mixed with 2 mL of distilled water and 2 mL of diluted Folin’s reagent. After 10 min, the absorbance of the blue color developed was measured spectrophotometrically at 760 nm. Total dry shoot phenol content was measured using the [107] procedure. In this procedure, 1 g of dry dried tomato shoots were extracted in 5–10 mL of 80% ethanol for at least 24 h. Alcohol was explained, the residual residue was re-extracted 3 times with 5–10 mL of 80% ethanol. Then, the clarified extract was filled to 50 mL with 80% ethanol. 0.5 mL of the extract mixed well with 0.5 mL of Folin’s reagent then shaken for 3 min. One milliliter of saturated Na_2_CO_3_ solution and 3 mL of distilled water were added and mixed well. After 1 h, the blue color was measured at 725 nm.

### 4.6. Estimation of Malondialdehyde (MDA) and Hydrogen Peroxide (H_2_O_2_) Contents

The content of MDA in fresh tomato leaf was measured according to [108]. Fresh leaf samples (0.5 g) were extracted with 5% trichloroacetic acid and centrifuged at 4000× *g* for 10 min. Two milliliters of the extract was mixed with 2 mL of 0.6% thiobarbituric acid (TBA) solution and was then put in a water bath for 10 min. After cooling, the absorbance of the developed color was measured at 532, 600 and 450 nm. Malondialdehyde content was determined using the following equation: 6.45 × (A532 − A600) − 0.56 × A450. The H_2_O_2_ content of fresh tomato leaf was measured as stated by [109]. In this method fresh tomato leaves (0.5 g) were added to 4 mL of cold acetone then 3 mL of the extract was mixed with 1 mL of 0.1% titanium dioxide in 20% (*v*:*v*) of sulfuric acid and the mixture was then centrifuged at 6000× *g* rpm for 15 min. The formed yellow color were measured at 415 nm.

### 4.7. Determination of Sodium (Na^+^) and Potassium (K^+^) Contents

Dry tomato shoot samples (0.1 g) were digested with 80% perchloric acid and concentrated sulfuric acid solution (1:5) for 12 h. The contents of Na^+^ and K^+^ in the digested samples were determined by flame photometry using the [110] method.

### 4.8. Assay of Antioxidant Enzymes Activity

Peroxidase (POD) activity was assayed according to that method described by [111]. The activity of polyphenol oxidases (PPO) was calculated by the procedure used by [112]. Superoxide dismutase (SOD) activity was determined by using a method described by [113]. Catalase (CAT) activity was assayed according to the method of [114]. The activities of POD, PPO, SOD and CAT were assayed in fresh tomato leaves.

### 4.9. Isozymes Electrophoresis

Native polyacrylamide gel electrophoresis (Native-PAGE) isozyme electrophoresis was performed to identify isozyme differences between control and treatment. PPO isozymes in leaf (100 mg fresh weight) samples were estimated as described by [115,116]. POD in fresh leaves isozymes were assessed by the procedure defined by [117]. SOD isozymes in fresh leaves were carried out as described by [118].

### 4.10. Protein Fingerprint

In the present study, tomato leaf protein fingerprints were analyzed using sodium dodecyl sulfate-polyacrylamide gel electrophoresis (SDS-PAGE) based on the method of [119], as modified by [120]. The molecular weight of proteins was then obtained relative to the marker, a large variety of molecular weight proteins (Gene Direx com).

### 4.11. Statistical Analysis

Two-way variance analysis (ANOVA) applied to the resulting data. Tukey’s honestly significant difference (HSD) using CoStat (CoHort, Monterey, CA, USA) was used to demonstrate statistically relevant differences between treatments at *p* < 0.05. Results are shown as mean ± standard errors (*n* = 3).

## 5. Conclusions

From the outcome of the obtained results, it could be concluded that foliar application of chitosan dissolved in different organic acids ameliorate the negative impact of salinity on tomato plants through enhancement of photosynthetic pigments and increasing osmoprotectant compounds, antioxidant system, potassium content and non-enzymatic system ROS scavenging. Therefore, it could be used in agricultural fields, especially those dissolved in ascorbic acid and citric acid, respectively. To the best of our knowledge, this is the first study explaining the impact of chitosan on isozymes and the protein fingerprints of tomato under salt stress and further molecular studies can stipulate information on the influence of chitosan on plant metabolism under salinity.

## Figures and Tables

**Table 1:** Effect of salinity (S) and foliar application of chitosan dissolved in different organic acids (Ch ACE, Ch ASC, Ch CIT, Ch MAL) and their interactions on the content of osmolytes (soluble sugars, soluble proteins and proline; mg g^−1^ DW) of tomato plants. Data are presented as means ± SE (*n* = 3). Data followed by different letters are significantly different following Tukey’s honestly significant difference (HSD) test at *p* ≤ 0.05. DW: Dry weight.

Treatments	Soluble Sugars	Soluble Proteins	Proline
Control	21.49 ± 0.24 e	15.37 ± 0.15 d	0.127 ± 0.001 f
Ch ACE	25.34 ± 0.19 c	21.87 ± 0.12 ab	0.118 ± 0.001 g
Ch ASC	28.67 ± 0.20 a	23.28 ± 0.09 a	0.116 ± 0.007 g
Ch CIT	26.49 ± 0.15 b	21.94 ± 0.05 ab	0.123 ± 0.001 f
Ch MAL	28.90 ± 0.16 a	22.45 ± 0.15 ab	0.118 ± 0.007 g
S	18.27 ± 0.26 h	9.72 ± 0.06 e	0.162 ± 0.002 e
S + Ch ACE	21.14 ± 0.20 ef	18.17 ± 0.08 cd	0.184 ± 0.001 d
S + Ch ASC	23.27 ± 0.19 d	20.63 ± 0.02 abc	0.237 ± 0.001 a
S + Ch CIT	20.11 ± 0.15 fg	18.13 ± 0.06 cd	0.191 ± 0.001 c
S + Ch MAL	19.94 ± 0.34 g	19.46 ± 0.04 bc	0.197 ± 0.002 b

**Table 2:** Effect of salinity (S) and foliar application of chitosan dissolved in different organic acids (Ch ACE, Ch ASC, Ch CIT and Ch MAL) and their interactions on the Sodium (Na^+^) and Potassium (K^+^) contents (mg g^−1^ DW) of tomato plants. Data are presented as means ± SE (*n* = 3). Data followed by different letters are significantly different following Tukey’s honestly significant difference (HSD) test at *p* ≤ 0.05. DW: dry weight.

Treatments	Na^+^	K^+^
Control	6.37 ± 0.02 e	1.42 ± 0.04 e
Ch ACE	7.15 ± 0.02 c	2.19 ± 0.01 c
Ch ASC	6.83 ± 0.01 d	2.35 ± 0.02 b
Ch CIT	7.37 ± 0.04 b	1.65 ± 0.01 d
Ch MAL	6.66 ± 0.03 d	2.49 ± 0.03 a
S	12.67 ± 0.06 a	0.728 ± 0.04 h
S + Ch ACE	6.20 ± 0.02 e	0.874 ± 0.01 fg
S + Ch ASC	5.29 ± 0.01 g	0.947 ± 0.01 f
S + Ch CIT	6.35 ± 0.02 e	0.805 ± 0.02 gh
S + Ch MAL	5.50 ± 0.03 f	0.884 ± 0.02 fg

**Table 3:** Isomers of peroxidase enzymes (+/−) and their Retention factor (Rf) in response to salinity Scheme. L1 = Control, L2 = Ch ACE, L3 = Ch ASC, L4 = Ch CIT, L5 = Ch MAL, L6 = S, L7 = S + Ch ACE, L8 = S + Ch ASC, L9 = S + Ch CIT and L10 = S + Ch MAL.

Rf	Treatments									
	L1	L2	L3	L4	L5	L6	L7	L8	L9	L10
0.189	+	+	+	+	++	++	+	+	+	++
0.246	−	−	−	−	+	+	−	−	+	+
0.393	+	+	+	++	++	++	++	++	++	++
0.533	++	++	++	++	++	++	+	+	++	++
0.762	+	+	++	++	++	+++	++	++	++	++
0.861	−	−	−	−	+	+	−	+	+	+
0.934	−	−	−	−	+	+	−	−	−	+

**Table 4:** Isomers of polyphenol oxidase enzymes (+/−) and their Retention factor (Rf) in response Table. L1 = Control, L2 = Ch ACE, L3 = Ch ASC, L4 = Ch CIT, L5 = Ch MAL, L6 = S, L7 = S + Ch ACE, L8 = S + Ch ASC, L9 = S + Ch CIT and L10 = S + Ch MAL.

Rf	Treatments									
	L1	L2	L3	L4	L5	L6	L7	L8	L9	L10
0.264	+	+	+	+	++	++	+	+	+	++
0.438	+	+	++	++	++	++	+	++	++	++
0.745	+	+	+	+	+	+++	+	+	+	+
0.847	−	−	+	+	+	++	−	−	+	+

**Table 5:** Isomers of superoxide dismutase enzymes (+/−) and their Retention factor (Rf) in response to salinity stress, foliar application of chitosan dissolved in different organic acids (Ch ACE, Ch ASC, Ch CIT and Ch MAL) and their interactions on tomato plants. L1 = Control, L2 = Ch ACE, L3 = Ch ASC, L4 = Ch CIT, L5 = Ch MAL, L6 = S, L7 = S + Ch ACE, L8 = S + Ch ASC, L9 = S + Ch CIT and L10 = S + Ch MAL.

Treatments										
Rf	L1	L2	L3	L4	L5	L6	L7	L8	L9	L10
0.362	+	+	+	+	+	+	−	++	++	−
0.641	+	+	+	+	+	+	+	+	+	+
0.727	−	+	+	+	+	++	++	++	++	−
0.865	+	+	+	+	+	+	+	+	−	+

**Table 6:** Protein electrophoretic banding patterns of salinity (S) and foliar application of chitosan dissolved in different organic acids (Ch ACE, Ch ASC, Ch CIT and Ch MAL) and their interactions of tomato plants. L1 = Control, L2 = Ch ACE, L3 = Ch ASC, L4 = Ch CIT, L5 = Ch MAL, L6 = S, L7 = S + Ch ACE, L8 = S + Ch ASC, L9 = S + Ch CIT and L10 = S + Ch MAL.

M.W KDa	Treatments										Polymorphism
	L1	L2	L3	L4	L5	L6	L7	L8	L9	L10	
116.221	+	+	+	+	+	+	+	+	+	+	Monomorphic
85.359	+	+	+	+	−	−	+	+	−	−	Polymorphic
72.115	+	+	+	+	+	−	+	+	+	−	Polymorphic
55.602	+	+	+	+	+	+	+	+	+	+	Monomorphic
43.487	+	+	−	−	+	+	+	+	+	+	Polymorphic
34.206	+	+	+	+	+	+	+	−	−	−	Polymorphic
28.165	+	+	+	+	−	−	+	+	+	−	Polymorphic
22.993	+	+	+	+	+	+	+	+	+	+	Monomorphic
17.779	−	−	+	+	+	+	−	+	+	+	Polymorphic
15.456	+	+	+	+	+	+	+	+	+	+	Monomorphic

## Data Availability

No new data were created or analyzed in this study. Data sharing is not applicable to this article.
